# Identification of dynamic driver sets controlling phenotypical landscapes^☆^

**DOI:** 10.1016/j.csbj.2022.03.034

**Published:** 2022-04-02

**Authors:** Silke D. Werle, Nensi Ikonomi, Julian D. Schwab, Johann M. Kraus, Felix M. Weidner, K. Lenhard Rudolph, Astrid S. Pfister, Rainer Schuler, Michael Kühl, Hans A. Kestler

**Affiliations:** a Institute of Medical Systems Biology, Ulm University, 89081 Ulm, Baden-Wuerttemberg, Germany; b Leibniz Institute of Aging – Fritz Lipman Institute, 07745 Jena, Thuringia, Germany; c Institute of Biochemistry and Molecular Biology, Ulm University, 89081 Ulm, Baden-Wuerttemberg, Germany

**Keywords:** Boolean network, Dynamic driver, Cellular phenotype control, Network dynamics, Intervention targets, Implicants

## Abstract

Controlling phenotypical landscapes is of vital interest to modern biology. This task becomes highly demanding because cellular decisions involve complex networks engaging in crosstalk interactions. Previous work on control theory indicates that small sets of compounds can control single phenotypes. However, a dynamic approach is missing to determine the drivers of the whole network dynamics. By analyzing 35 biologically motivated Boolean networks, we developed a method to identify small sets of compounds sufficient to decide on the entire phenotypical landscape. These compounds do not strictly prefer highly related compounds and show a smaller impact on the stability of the attractor landscape. The dynamic driver sets include many intervention targets and cellular reprogramming drivers in human networks. Finally, by using a new comprehensive model of colorectal cancer, we provide a complete workflow on how to implement our approach to shift from *in silico* to *in vitro* guided experiments.

## Graphical abstract

**Figure:**
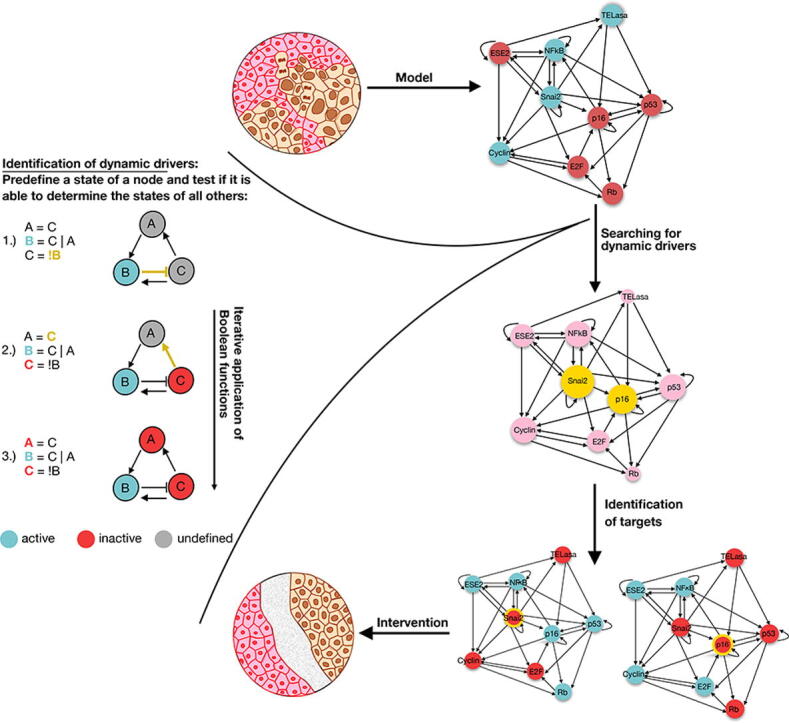

## 1 Introduction

Modern biology has shifted toward investigating complex regulatory networks and their dynamic behavior [1]. Hence, network analysis has emerged as a powerful tool to understand molecular crosstalk [2]. Here, compounds of the biological system are represented as nodes in the network models. By this, diseases such as the development of cancers are rarely a consequence of a mutation of a single component within a network but rather of its global perturbation [2], [3]. Thus, to understand any biological process, we need to capture the network dynamics [4]. In the case of biomolecular regulatory networks, especially Boolean networks, this identifies the stable states of a system [5] that can mathematically be defined as attractors. Attractors correlate with biological phenotypes, e.g., cellular proliferation [6], [7], [8], death [9], [10], or differentiation [11] based on the activity of some compounds or final phenotypical states. Activity patterns in attractors can also be validated by comparison to experimental results [7], [9], [10], [12]. However, unraveling the full dynamics is a complex and demanding task. Therefore, several studies have attempted to identify small sets of dynamic drivers able to control the shift towards a specific phenotype based solely on the network structure [13], [14]. These structure-based approaches are quite limited when applied to biomolecular networks. This is mainly because they assume linear dynamics and time-varying control of nodes, which are unfeasible in biological regulatory networks [13], [14]. Kim and colleagues [5] proposed a method to identify ‘kernels’ responsible for shifting network dynamics toward the primary stable state (attractor) [15], [16]. Even if their work focuses on network dynamics, still the control of the kernel set determines only single attractors and not the complete landscape of possible ones [5].

In contrast, one might be interested in knowing whether there exists a small set of nodes that is sufficient to determine all possible cellular behaviors described in the network. In this case, the ability to control a given phenotype is lost for inferring a minimal set responsible for the entire network dynamics. This would be advantageous when the network size is too large to allow in-depth dynamic analyses or when knowledge of desired attractor patterns is missing. In this context, by knowing this minimal set of dynamic drivers, it would be possible to reconstruct cellular phenotypes. Moreover, in principle, these dynamic drivers could be targeted independently for a wide range of intervention studies.

In the present work, we investigated whether the whole landscape of cellular behaviors can be controlled by a minimal set of nodes of the underlying biomolecular network. Following previous studies, we applied logic-based Boolean network models that have the main advantage of avoiding the use of kinetic parameters that are often not available in the biological research [17]. We identified small sets of nodes that alone are sufficient to retrieve the complete phenotypic landscape of the system. We analyzed 35 published Boolean networks and developed a heuristic algorithm for identifying these sets of dynamic driver nodes.

Considering the total number of nodes in the analyzed networks, we found that the identified sets cover a small fraction of the complete network and depend on specific topological features. We further studied the applicability of targeting these dynamic drivers independently. An ideal intervention target is expected to shift dynamic behaviors towards a desired effect on the phenotype without producing side effects, such as increased instability to the system or resistant phenotypes. We translated this concept by extensively studying *in silico* single node perturbations over all our selected networks. The identified dynamic drivers significantly impact shifting dynamics without causing an insurgence of further attractors. This was confirmed in the analyses of three *in silico* case studies concerning both intervention targets and cellular reprogramming. Finally, we introduce a new model and provide a complete workflow endowed with *in vitro* experiments on how to apply the presented method for drug targeting purposes. Thereby, we show a complete operative example from simulation to bench procedure.

## 2 Materials and methods

### 2.1 Boolean networks

In Boolean networks, nodes are described as Boolean variables $X=\left\{x_{1},\cdots ,x_{n}\right\},x_{i}\in B$ representing compounds within the system. Each can be assigned to a state of 1 (expressed/active) or 0 (not expressed/inactive). Boolean functions represent biochemical reactions $F=\left\{F_{1},\cdots ,F_{n}\right\},F_{i}:B^{n}\to B$ [18], [19]. If the value of $F_{i}$ depends on $x_{i_{1}}$, $x_{i_{2}},\cdots ,x_{i_{k}}$ let $f_{i}$ denote the function defined on these inputs, i.e. $F_{i}\left(x\right)=f_{i}\left(x_{i_{1}},x_{i_{2}},\cdots ,x_{i_{k}}\right)$. $f_{i}$ is also called the Boolean function for the i-th position (e.g. gene) of F. In our modeling approaches; we model input nodes via the identity function. This ensures the input is constant once set. To analyze the dynamics of Boolean networks over time, the state of the networks $x\left(t\right)=\left(x_{1}\left(t\right),\cdots .,x_{n}\left(t\right)\right)$ is defined by the states of all variables $x_{i}$ at a point in time *t*. In synchronous Boolean network models, all Boolean functions are updated at the same time to proceed from the state at time *t* to its successor state at time *t + 1,* which is defined by $x\left(t+1\right)=\left(F_{1}\left(x\left(t\right)\right),\cdots ,F_{n}\left(x\left(t\right)\right)\right)$. $x\left(0\right)$ is the initial state of the network. The dynamics of Boolean network models can be viewed in a state transition graph linking each state of the state space to its successor state. The state-space of Boolean networks with *n* nodes is finite with $2^{n}$ possible states. Thus, the model will eventually enter a recurrent sequence of states called attractors (cycles), depicting the long-term behavior of the network. A network can have more than one attractor. In this case the final sequence of states (attractor) depends on the initial state. In a biological context, attractors are associated with phenotypes.

All Boolean network simulations were performed with R v3.4.4 [20] and the R-package BoolNet [21] v2.1.5.

### 2.2 Boolean network model selection

For our analysis, we extracted Boolean functions of Boolean network models from PubMed with the search item “Boolean network model” as well as Boolean functions from the Interactive modelling of Biological Networks database [22] (https://cellcollective.org). Networks were selected until May 24th, 2017. We excluded networks whose dynamics could not be investigated in feasible time, networks with too many attractors, and networks in which nearly all nodes are input nodes. Additionally, we excluded non-scale-free networks and those that could be reduced to their input nodes. We analyzed 35 networks with sizes ranging from 5 to 40 nodes.

### 2.3 Test for scale-freeness

If a Boolean network has a scale-free network architecture, it can be described by the power-law distribution $P\left(k\right)\propto k^{-\alpha }$ where α is the power-law scaling parameter and *k* is the number of links in the network. To identify scale-freeness, we tested if the power-law distribution can plausibly describe the network's degree distribution by using the R-package poweRlaw v.70.2 [23].

### 2.4 Determination of the minimal dynamic node-set

Two strategies were applied to determine a minimal set of nodes determining the dynamics of the complete network. The heuristic is defined in terms of significance. Here, the importance of a node is maximal if it is a constant or an input node, which we modeled using the identity function only depending on its own value. Otherwise, the significance of node *g* is equal to the number of nodes whose transition function depends on *g*. Therefore, the heuristic selects a node g with the highest significance in each iteration until a set of dynamic driver nodes is found (Appendix Method A.1). For reference, we use an exhaustive search algorithm to find minimal dynamic driver sets *G* of size *k* for increasing values of *k*. The algorithm terminates for the smallest value of *k* such that some subset $G:G=g_{i1},\cdots ,g_{\mathrm{ik}}$ is a dynamic driver set, i.e. for every assignment a network state is observable (Appendix Method A.2).

### 2.5 Reducing network size

The complexity of Boolean networks increases rapidly with each additional node, and exhaustive search methods face some difficulty in being be applied to more extensive networks. Therefore, we reduced the search space of large Boolean networks to accelerate the analysis. This was achieved by iteratively removing nodes that do not regulate other nodes (outputs). The procedure was repeated until all superfluous nodes were removed (Appendix Method A.1). Please note that the network reduction was only applied to search for dynamic drivers. This is because output nodes, by definition, have no outgoing links and thus do not influence the rest of the nodes of the model (see Appendix Methods A.1–A.2). In contrast, all other performed measures strictly depend on the network topology. Hence, to avoid alteration of the original network structures, all further investigations were performed on the entire network.

### 2.6 Partial assignments

**Fig. 1:**
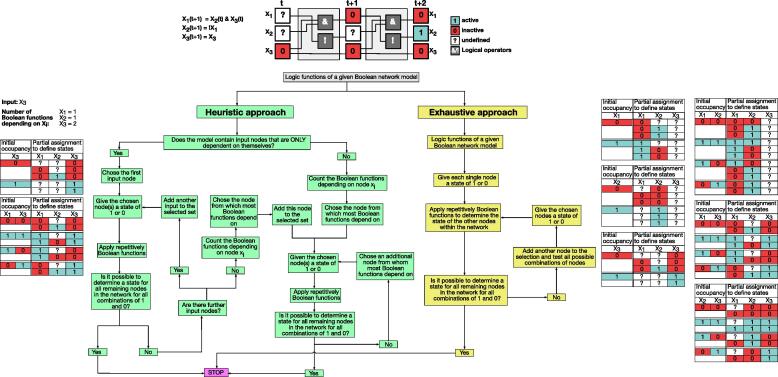
Identification of dynamic drivers. The upper part shows a toy model consisting of three nodes (x_1_, x_2_, x_3_) and corresponding regulatory logic functions. The update scheme is represented as a circuit, and two-time steps are depicted, which are required to determine the entire states of the model based on the predefined driver (here x_3_). Colors and symbols are explained in the legend. Below is a complete logic workflow of the two implemented approaches to identify dynamic drivers. On the left, in green, is shown the heuristic approach. The exhaustive one is depicted on the right in yellow. Finally, operative examples based on the toy model are displayed on both sides of the flow chart for each approach. (For interpretation of the references to colour in this figure legend, the reader is referred to the web version of this article.)

A partial assignment defines the value of some nodes of the Boolean network. The value of the other nodes is undefined. The transition function $F=\left\{F_{1},\cdots ,F_{n}\right\},F_{i}:B^{n}\to B$ can be applied to a partial assignment as follows: if the value of the transition function $F_{i}$ of a node *i* is uniquely determined from nodes which are defined under the partial assignment, then the node is set to this value. The value of all other nodes remains unassigned initially (Fig. 1). Repeated application of the transition function to a partial assignment will define the value of not necessarily all nodes. If the assignment can be extended to all nodes and hence defines a network state, we say that the network state is observable from the partial assignment. A set of nodes is called a dynamic driver if a network state is observable for every assignment to the nodes. A dynamic driver set is minimal if the cardinality of the set is minimal.

### 2.7 Connectivity

Their incoming and outgoing edges define the static connectivity of nodes within a Boolean network. To identify static hub nodes, we standardized connectivity using the z-transform: $Z=\frac{K_{i}-K_{n}}{\sigma }$ whereby $K_{i}$ is the number of interactions of component *i* to any node in the network, $K_{n}$ is the average of interactions of all compounds within the network and σ is the standard deviation of $K_{n}$. Compounds with a z-score > 2.5 are considered hub nodes [24].

### 2.8 Analysis of total biological interactions

Protein interaction tables of each organism considered in one of our analyzed Boolean network models were obtained from BioGRID [25] (Version 3.5.168), and the directed interactions of each protein were counted with R [20]. In the case of Boolean network nodes consisting of several proteins or nodes whose proteins can be several isoforms, the average of all these possible proteins was taken. Afterward, the z-score was used again to define biological hubs.

### 2.9 Analysis of essential genes

We used the HEGIAP database [26] to identify essential genes. Essential genes are organism and context-dependent. For this reason, the database provides information on essentiality in *H. sapiens, M. musculus, S. cerevisiae, D. rerio, S. pombe, and D. melanogaster*. Given that most of the models in our selection deal with human-related regulatory processes, we extracted essential genes for *H. sapiens*, which were included in our selection of Boolean network models. The number of essential genes was compared to our dynamic drivers.

### 2.10 Network diameter

For all networks, we analyzed the changes in network diameter upon removing single nodes, accounting for the directionality of edges, using the diameter(graph, directed = TRUE, unconnected = TRUE) function from the R-package igraph v1.2.6 [26].

### 2.11 Canalyzation

A node is said to canalyze a Boolean function if knowledge of the state of this node is sufficient to determine the function’s output [27]. For every node in a given Boolean network, we counted the number of Boolean functions for which it acts as a canalyzing node, which we call the canalyzation number.

### 2.12 Comparison to ‘kernel nodes’

Kim and colleagues [5] published a method to identify ‘kernel nodes’ as dynamic drivers and tested their method on Boolean network models. Given that this approach is attractor-dependent, we considered the union of kernel nodes for all attractors. We compared the overlap of identified ‘kernel nodes’ to our dynamic drivers.

### 2.13 MAPK/Wnt model establishment

Key MAPK-signaling and canonical Wnt-signaling cascade components and their crosstalk components were considered for the model setup.

The model was manually curated based on a literature search [28]. Here, first main components of the investigated crosstalk were evaluated and included via a Google Scholar and PubMed search. Interactions were included, prioritizing results driven from the CRC context, integrating both *in vitro* and *in vivo* information. When available, also interactions observed in patients-derived tumoral tissues were included. Different regulatory levels were also considered, ranging from regulation of expression to protein alterations. Finally, interactions were refined via screening of curated databases BioGRID [25] and Metacore^TM^ (Thomson Reuters Inc., Carlsbad, CA). A detailed description of the model setup rationale and dynamic analysis is available in the Appendix Method A.4.

### 2.14 Mammalian cell line and cell culture

SW480 cells initially derived from a 50 years old male with colorectal adenocarcinoma were obtained from the American Type Culture Collection (ATCC) to ensure authentication. Cells were cultured at 37 °C and 5% (v/v) CO_2_ in DMEM high glucose (Sigma-Aldrich) medium containing 10% fetal bovine serum (Life technologies) and 1% Penicillin-Streptomycin (Life technologies). Cells were routinely tested for the absence of mycoplasma (GATC).

### 2.15 Drug treatment

Drug treatments were performed in 6-well plates 24 h after seeding; cells were treated with 1 mL of medium containing 2 µM BVD-523 (HY-15816, MedChemExpress) or 12 µM TD-52 (SML2145, Sigma-Aldrich) or a combination of BVD-523 and TD-52. Drugs were dissolved in DMSO (Sigma-Aldrich).

### 2.16 Proliferation and apoptosis assay

10 µL of cells were mixed with Trypan blue (Invitrogen), loaded on a cell counting slide (Countess^TM^, Invitrogen), and counted with the Counter II (Invitrogen). Here, we also detected the amount of living viable and dead cells.

### 2.17 Wound healing assay

Cells were seeded on fibronectin-coated coverslips. After cells were confluent, they were treated as described before. The wound was introduced with a 200 µL pipette tip. A series of pictures of each wound was taken (BIOREVO BZ-9000, KEYENCE, magnification 4x) and merged (program BZ analyzer II, KEYENCE) to a complete photo of the wound that was analyzed further. Wound closing was measured by calculating whole wound border areas at different time points for each repetition with the ‘MRI wound healing tool’ implemented in Image-J [29].

### 2.18 Immunofluorescence staining

SW480 cells were grown on glass coverslips. Cells were fixed with 4% paraformaldehyde (PFA, Carl Roth) for 15 min and were permeabilized with 0.1% Triton X-100 (Merck) for 10 min. Cells were blocked in 0.5% bovine serum albumin Fraction V (BSA, cytiva) for 45 min, incubated with primary (E-cadherin #610181, BD Bioscience, 1:200) and secondary antibodies (anti-mouse Alexa 488, dianova, 1:1000) for 2 h and 1 h at RT each, and were mounted in DAPI mounting medium (Invitrogen). Using LAS AF software, images were taken with a Leica TCS SP5 II confocal microscope in a single plane (63-objective). Same exposure settings were used in controls and drug-treated samples.

Images of the same experiment were processed equally using Adobe Photoshop CS6 software.

### 2.19 Drug target and resistance analysis for the CRC model

The nodes of the CRC model have also been screened for targeted therapeutic approaches in cancer therapy. Here, we focused on targeted approaches of any kind (small molecules, siRNA, vaccines etc.) that have reached clinical trials (at least one). The screening was performed by searching the clinicaltrails.gov website and on the Therapeutic Target Dataset [30], with the results collected on the 1st of March 2022. Additionally, clinically employed targets have also been investigated for an insurgence of resistance to therapy in treated patients via a literature-based screening. For this analysis, some nodes have been excluded since they refer to helper or output nodes (Destruction complex (DC), Tight Junctions (TJ), SCF (scaffold)). Finally, for GSK3β, different activities are represented by nodes in the model, summarized in one node for this search.

### 2.20 Quantification and statistical analysis

Analyses and visualization were done with R [20] (https://www.r-project.org). All statistical tests are two-sided.

**Fig. 2:**
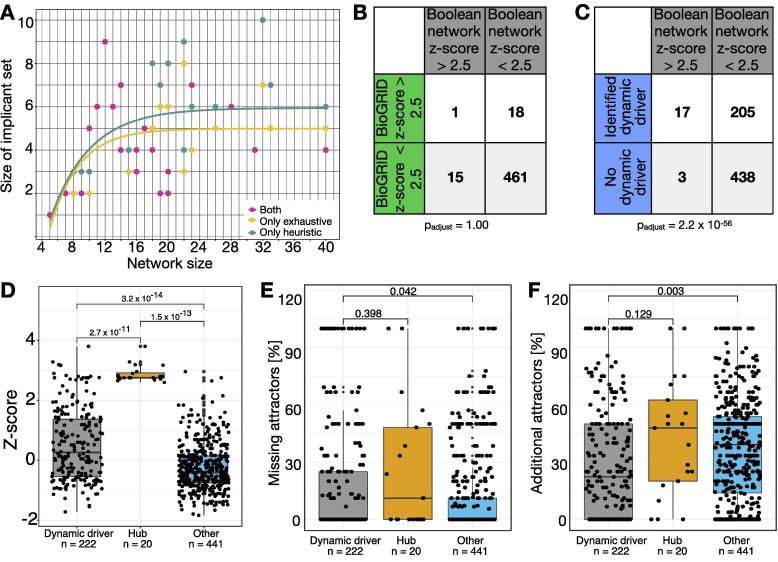
Dynamic *in silico* analyses. (A) The dynamic drivers' overall set size is small and independent of the network size (best fit with logarithmic fit). (B) Regulatory interactions of nodes in Boolean network models are comparable to their biological representatives. (C) The majority of dynamic drivers are no hub nodes. Nodes are defined as hubs if their z-score was > 2.5 [24]. Statistics were performed with Cochran’s Q test with a post-hoc pairwise sign test with Bonferroni correction. (D) Distribution of z-transformed connectivity among dynamic drivers, hubs, and other nodes. (E) Percentage of missing attractors or (F) additional attractors after interventions (knockouts/overexpression; Wilcoxon test). We adjust p-values via Bonferroni corrections and assume significant results if p < 0.05. p-values are depicted on top of each comparison bar.

Fig. 2B/C: Statistics were performed with Cochran’s Q test and post-hoc pairwise sign test with Bonferroni correction (R package RVAideMemoire [31]).

Fig. 2D-F: Effects of interventions and z-scores were analyzed by Wilcoxon test (dynamic drivers vs hubs/other nodes) with Bonferroni-correction.

Fig. 4A-C: Experimental data were analyzed by Wilcoxon test (each treatment vs untreated).

## 3 Results

In the following, we will use our method to identify a set of nodes able to determine the entire dynamic behavior. We applied our approach to a collection of biologically motivated Boolean models and obtained the dynamic drivers accordingly. Given that biologically relevant genes have been connected to some network properties (such as connectivity level), we further investigated our sets in this regard. Biologically relevant nodes are also supposed to yield significant effects when perturbed. Hence, we analyzed the behavior of our sets when perturbed from both a network dynamic and a biological relevance perspective. Finally, having confirmed the relevance of our dynamic drivers, we applied our method for investigating new targeted approaches (both *in silico* and *in vitro*) in a newly established CRC model.

### 3.1 Identification of dynamic drivers

In biomedical research, Boolean networks generally model biologically relevant compounds and their interactions to capture a specific process. Given that this modeling approach allows for evaluating the system's dynamic, it is of great interest to develop methods to identify efficient disease drivers and targets within the modeled crosstalk. Hence, previous *in silico* studies already showed that small sets of compounds are sufficient to control the shift of the network dynamics towards the desired attractor independently from the initial starting conditions [5], [32]. Exemplarily, one might be interested in identifying compounds responsible for the shift from a quiescent to cancer related attractor or from a young to an aged one (e.g., models from [8], [33]). However, this strategy implies a previous knowledge of the attractor landscape and a potential interpretation of the system's dynamic behavior. Here, we want to define the minimal set of compounds sufficient to determine the full dynamics, thus reaching all attractors of the examined model. Following prior approaches [34], [35], [36], we used 35 logic-based Boolean network models (Table 1) for our investigations. This model type belongs to one of the simplest dynamic models where molecular compounds are represented as nodes (n), and their interactions are summarized in Boolean functions (*f*). Nodes can be either active or inactive. These states are represented by binary values (0 for inactive, 1 for active). Dynamic simulations are performed by recursively applying Boolean functions for each node until a steady-state is reached. Under the synchronous update assumption, each node is updated by applying its Boolean function at each discrete step in time. Therefore, the state of a Boolean network is given by a vector of values (0/1) assigned to their nodes. Thus, a state change results from applying a Boolean function for each node [18]. Hence, the state change of a node depends on the previous state of other nodes. This state change of previously not encountered states will occur until an attractor is reached. Keep in mind that attractors can be associated with biological phenotypes based on the activity of several nodes. In the following, we always intend phenotypes in terms of attractors and phenotypical landscape in terms of attractor landscape. Based on previous results, we assume that not all nodes are involved in the network's dynamic behavior. Therefore, we implemented an approach to identify sets of dynamic drivers from which it is possible to determine all states of a network.

**Table 1:** Boolean network models investigated Depicted are the described process and the organism for which the model was set up.

Network	Process	Organism
Azpeitia et al. [37]	Root stem cell niche	*Arabidopsis thaliana*
Brandon et al. [38]	Oxidative stress response	*Aspergillus fumigatus*
Calzone et al. [39]	Cell-fate decision	*Homo sapiens*
Cohen et al. [40]	EMT	*Homo sapiens*
Dahlhaus et al. [6]	Cancer signaling in neuroblastoma	*Homo sapiens*
Davila-Velderrain et al. [41]	Early flower development	*Arabidopsis thaliana*
Enciso et al. [42]	Lineage fate decision of hematopoietic cells	*Homo sapiens*
Fauré et al. [43]	Mammalian cell cycle	*Homo sapiens*
García-Gómez et al. [44]	Root apical meristem	*Arabidopsis thaliana*
Giacomantonio and Goodhill [45]	Cortical area development	*Homo sapiens*
Gupta et al. [46]	Neurotransmitter signaling	*Homo sapiens*
Herrmann et al. [11]	Cardiac development	*Homo sapiens*
Irons [47]	Cell cycle	*Saccharomyces cerevisiae*
Klamt et al. [48]	T-cell receptor signaling	*Homo sapiens*
Krumsiek et al. [49]	Hematopoiesis	*Homo sapiens*
MacLean and Studholme [50]	Type III secretion system	*Pseudomonas syringae*
Mai and Liu [51]	Apoptosis	*Homo sapiens*
Marques-Pita and Rocha [52]	Body segmentation	*Drosophila Melanogaster*
Marques-Sanchez et al. [53]	CD4 + T-cell fate	*Homo sapiens*
Méndez and Mendoza [54]	B-cell differentiation	*Homo sapiens*
Méndez-López et al. [33]	Immortalization of epithelial cells	*Homo sapiens*
Mendoza and Xenarios [55]	T-cell signaling	*Homo sapiens*
Meyer et al. [9]	Senescence-associated secretory phenotype	*Homo sapiens*
Orlando et al. [56]	Cell cycle	*Saccharomyces cerevisiae*
Ortiz-Gutiérrez et al. [57]	Cell cycle	*Arabidopsis thaliana*
Ríos et al. [58]	Gonadal sex determination	*Homo sapiens*
Saadatpour et al. [59]	T-cell large granular lymphocyte survival	*Homo sapiens*
Sahin et al. [60]	Cell cycle	*Homo sapiens*
Sankar et al. [61]	Hormone crosstalk	*Arabidopsis thaliana*
Siegle et al. [8]	Aging of satellite cells	*Homo sapiens*
Sridharan et al. [62]	Oxidative stress	*Homo sapiens*
Sun et al. [63]	Endomesoderm tissue specification	*Sea urchin*
Thakar et al. [64]	Immune response	*Homo sapiens*
Todd and Helikar [65]	Cell cycle	*Saccharomyces cerevisiae*
Yousefi and Dougherty [66]	Metastatic melanoma	*Homo sapiens*

In other words, by only knowing the state of the set of the dynamic drivers, it is possible to infer all the other states by just applying iteratively Boolean functions (see Fig. 1, upper panel). We implemented two strategies to search for a minimal set of dynamic drivers k (Fig. 1). The pseudocode is provided in the Appendix Methods A.1–A.2. The exhaustive approach computes every possible combination of potential candidate drivers until the minimal set for dynamic inference is defined (Fig. 1, Exhaustive approach depicted in yellow and Demo available on the git repository). This approach is prohibited in large networks due to the exponential increase of complexity and time with network size ($O(n^{k}∙2^{k})$). Thus, we applied the full search only as a reference to evaluate our newly established heuristic search algorithm. The latter computes growing sizes of driver sets until the set is sufficient to determine all other states of the nodes in the network (Fig. 1, Heuristic approach depicted in green Demo available on the git repository). For this purpose, the heuristic approach assigns a weight to each node. Starting from external inputs, the algorithm iteratively considers additional potential drivers based on a descending number of Boolean functions depending on this node (Fig. 1). In the following, we will analyze and characterize the resulting dynamic driver sets from our set of logic models (Table 1).

### 3.2 The minimal set of dynamic drivers is small, and its size correlates with neither topological nor dynamic properties

First, we compared the performance of the heuristic and exhaustive approach applying our method to our set of networks (Table 1). Comparing the performance of both algorithms, we found that the heuristic approach identified a minimal set of dynamic drivers for 54.3% of the networks. Furthermore, in 32 out of the 35 networks considered, the set of dynamic drivers defined by the heuristic was a superset of the minimal set found by an exhaustive search. Hence, solutions from the exhaustive approach are all found also by the heuristic one. This means the heuristic method correctly identifies the minimal set; however, it might add further nodes not strictly required. The performance of the heuristic is comparably favorable considering the required run times complexity of $O(n^{2}∙k∙2^{k})$.

Furthermore, our analyses revealed that the typical size of the minimal set of dynamic drivers is small (Fig. 2A). The cardinality of a set of dynamic drivers ranges from 1 to 9 for an average network size of 19 nodes. Here, the exhaustive approach identified a mean of 4.4 dynamic drivers within a set of nodes and the heuristic a mean number of 5.1 nodes.

We investigated if the dynamic driver set size is affected by topological or dynamic features. Only a low correlation between the network size and the size of dynamic driver sets (Pearson correlation: r_exhaustive_ = 0.32, r_heuristic_ = 0.39), as well as between the size of dynamic driver sets and the number of attractors (Pearson correlation: r_exhaustive_ = 0.58, r_heuristic_ = 0.50) could be found. We further found a poor correlation between the size of our sets of dynamic drivers and the number of inhibitory regulations in the networks (Pearson correlation: r_exhaustive_ = 0.46, r_heuristic_ = 0.55). Similar results were observed using a linear fit (network size ∼ dynamic drivers: R^2^_exhaustive =_ 0.10, R^2^_heurisitic =_ 0.15; attractors ∼ dynamic drivers: R^2^_exhaustive =_ 0.30, R^2^_heurisitic =_ 0.20; inhibitory regulations ∼ dynamic drivers: R^2^_exhaustive =_ 0.21, R^2^_heurisitic =_ 0.30) indicating that the size of the dynamic driver set does not increase linearly with the network size, the number of attractors, or the number of inhibitory interactions in the networks. Instead, we obtained the best fit using a logarithmic function for what concerns the network size (Fig. 2A).

### 3.3 The identified set of dynamic drivers is different from the ones identified by previous approaches

Different studies have suggested dynamic influencing nodes based on topological or dynamic features [19], [67], [68], [69], [70], [71]. For this reason, we investigated whether our set of dynamic drivers is univocally identifiable or overlapping with sets suggested by other authors.

From a topological perspective, it is well established that highly connected nodes, defined as hubs, impact the network dynamics [68]. Therefore, we studied the connectivity of the sets of dynamic drivers to assess if they are mainly hub nodes. First, we considered that the number of links found in Boolean networks might represent only a subset of the real regulatory interactions. This could in principle alter the identification of hub nodes. Hence, we compare the hubs identified in the Boolean models with the ones identified by considering the number of links from BioGRID [25]. Here, we did not find any differences between the degree of connectivity in Boolean models and the BioGRID database (p = 1.0). As a consequence, we could show that the level of connections represented in the Boolean network models for each node faithfully represents the one in protein–protein interaction networks scenarios (Fig. 2B).

Across all considered Boolean network models (Table 1), only 3.2% of nodes could be identified as hubs (z-score > 2.5 [24]). Comparing these nodes with our sets of dynamic drivers showed that they differ significantly (p = 2.2 ∙ 10^-56^) (Fig. 2C). This is in accordance with the distribution of z-scores (Fig. 2D), which is significantly lower for dynamic drivers than hubs. According to other studies, the high impact of hub nodes might relate to their potential role as essential genes. In this context, it could be shown that only 20% of hubs are essential genes [72] for human networks. Being aware that the definition of essentiality is context-dependent, we quantified the proportion of essential genes being dynamic drivers or hubs in our set of human networks. By screening the HEGIAP database [72], we found that 12% of our nodes were classified as essential in humans. Of these, 27.5% are dynamic drivers, and only 4% are hubs. Moreover, all essential hubs identified were also dynamic drivers.

As a second topological measure, we investigated whether our set of dynamic drivers coincides with nodes whose removal coincides with an enlargement of the networḱs diameter [71]. Again, our selected set of drives does not significantly induce diameter changes (Appendix Fig. A1 A, p = 0.58). Accordingly, only 34% of the total dynamic driver set causes a diameter shift. In contrast, highly connected nodes strongly induce changes in network diameter if compared to both dynamic drivers and other nodes (Appendix Fig. A1 A, p ≤ 0.009), and 65% of hubs are also causing a diameter shift.

In summary, our set of selected dynamic drives is not identifiable by topological measures relevant to influencing the networḱs dynamics.

We compared our dynamic driver sets to dynamic features known to influence network behavior as a next step. The depth of canalizing functions is known to affect the stability of networks by reducing the number of attractors (phenotypes) and avoiding unstable behaviors (large cycling states) [19], [70]. Therefore, canalization is, in general, a desired dynamic property of biological networks. First, we analyzed the distribution of canalyzers overall analyzed networks. Here, we found that in our group of networks, 78% of nodes canalyze at least one function, accounting for the biological relevance of the selected regulatory networks.

Nevertheless, only 10% of nodes act as canalyzing nodes in more than three functions, given a general average canalization number of 1.6 overall analyzed networks. Among these, more than half of the nodes are also found in the selected dynamic driver sets from our approach. In general, we could show that our selected set of dynamic drivers shows a higher presence as canalyzing nodes when compared to the rest of the nodes (Appendix Fig. A1 B). Nevertheless, hub nodes still score a significantly higher number of canalized functions. The presented results show that our approach includes also but not only, a large amount of highly canalizing nodes. This accounts for the relevance of our approach, still intriguingly showing that our method identifies a set of nodes independent from other approaches.

Besides investigating the topological and dynamic features relevant in biologically motived networks, we also compared our results to another well-established method to derive dynamic drivers. Kim and colleagues published a method to identify ‘kernel nodes’ which are applied in the context of control of single network phenotypes in logical models. Therefore, we compared the results of our method in the networks that were commonly analyzed in both works. By comparing the resulting sets, we found that they only partially overlap. Besides, there is no general tendency indicating that our sets might be either a super- or a subset of the kernel sets from Kim and colleagues [5]. This difference in the results might relate to the necessity of controlling a different set of nodes to direct the systems towards only one desired phenotype. In other words, retrieving one attractor might require different specifications in terms of activity knowledge compared to the entire set of attractors.

### 3.4 The perturbation of single dynamic drivers alters network dynamics and provides biologically relevant interventions

We showed that our method identifies minimal sets of nodes able to resume the dynamic landscape in terms of attractors of a certain network. Since these nodes are so relevant to the dynamic behavior of the examined models, they might also be interesting perturbation targets able to efficiently alter the long-term behavior of the analyzed system *in vitro* or *in vivo*. In the concepts of logic modeling, permanent fixation of components to either 1 or 0 can be compared to *in vitro* overexpression or knockout experiments. This can be tackled for single as well as for multiple nodes simultaneously. In control theory applied to biological networks, driver sets need to be all altered to shift behaviors [5], [69]. However, it might be difficult if not infeasible to control more than one or two nodes simultaneously, especially thinking of interventions applicable to the clinical context. Hence, we investigated whether altering one single element in our sets can still perturb the dynamic behavior of the system.

Thus, we performed perturbation experiments with both overexpression and knockout of all 663 nodes present in our selection of Boolean networks. The perturbed attractor sets were compared to the originally retrieved attractors in the unperturbed conditions. By matching the attractors’ activities, we could evaluate if the set of perturbed attractors presented a gain or loss of some of the attractors in the dynamic landscape (Fig. 2E-F). Both losses and gains of attractors are considered as having a high impact on the dynamic landscape of the investigated models. However, the gain of new attractors can also indicate a decrease in the stability of the investigated system. For example, when considering an intervention target for drug targeting purposes, the emergence of a new attractor can make the dynamic landscape more heterogeneous and difficult to evaluate. In addition, the new attractors might also report activities connected to side effects or resistance. Finally, we grouped our nodes based on being assigned to the dynamic driver set or not. Further, hub nodes were used for comparison control as known inducers of network long-term activities. Our results show that a single perturbation of genes belonging to the dynamic driver sets yields significantly higher effects than the rest of the nodes and is comparable to hub nodes (Fig. 2E-F). Interestingly, additional attractors are also significantly reduced if single dynamic drivers are perturbed (Fig. 2F). Overall, we provided evidence showing that a single perturbation of nodes belonging to the dynamic driver sets significantly affects attractors’ landscapes, potentially evoking fewer alterations in the stability of the investigated system.

To add a further layer of biological interpretation to the significant perturbations observed in the attractor landscapes as suggested in Ikonomi et al. [28], we further analyzed and interpreted the obtained attractor patterns in three case studies from our set of analyzed models. Matching the *in silico* prediction with a biological phenotype is crucial in the final evaluation of an attractor landscape. Hence, the resulting attractors’ activities from the perturbed conditions were compared to the experimental results of published studies.

Cohen et al. [40] describe molecular pathways of tumor development to invasion and metastases. In their network, we identified AKT2 and TWIST1 as dynamic drivers. While the simulation of AKT2 overexpression reached attractors supporting tumor development by inhibiting apoptosis and activation of epithelial to mesenchymal transition (EMT), *in silico* knockout of TWIST1 prevents tumor-associated characteristics. Our *in silico* results are supported by the literature. Here it is described that AKT2 mediates EMT by inhibiting GSK3β/Snail signaling [73] and that its overexpression in combination with PTEN loss promotes metastases [74]. Both events thus support tumor formation. In contrast to the negative effect of AKT2, the favorable effect of TWIST1 could also be confirmed by a literature search. A knockout of TWIST1 in breast cancer cells inhibited the expression of EMT markers and prevented metastases in immune-deficient mice [75].

Likewise, the network of Méndez-López et al. [33] captures the EMT process. Here, all identified dynamic drivers (Snai2, ESE2, and p16) are correlated to strong effects on the phenotypic landscape. Nevertheless, our *in silico* perturbations suggest that the strongest intervention effect can be observed by targeting the dynamic driver node Snai2. While the unperturbed network simulation ends in three single state attractors representing epithelial, senescent, and mesenchymal characteristics [33], the simulation of Snai2 overexpression only yields one attractor with mesenchymal characteristics [33]. The attractor with mesenchymal characteristics disappeared by simulating Snai2 knockout [33]. Laboratory experiments also support these effects of Snai2 in the Boolean network model. Here, *in vitro* overexpression of Snai2 resulted in a mesenchymal appearance of cells within 72  h [76] while depletion of Snai2 supports premature differentiation [77].

As a final case study, we presented a case study in the context of intervention for cell reprogramming. The Boolean network of Krumsiek et al. [49] describes hematopoietic stem cell differentiation. Based on our analysis, we identified six dynamic driver nodes. A knockout of each of these proteins leads to the loss of a blood cell lineage in the simulation, while abnormal states are absent [49]. This is in line with results from *in vitro* experiments [78], [79], [80].

To sum up, literature comparison of our simulations of interventions in three different case studies could enforce our results independent of the cell context. Interestingly, none of the presented case studies included hub nodes in the selected sets. Based on these results, it can be reasoned that even nodes with only a few connections in a network structure can change the phenotype of biological processes. Overall, we provide a method to efficiently determine biologically motivated intervention targets in logic-based models.

### 3.5 Moving from simulation to laboratory validation: A workflow on how to apply the method to identify new potential drug targets

Systems biology provides a holistic view of complex regulatory processes, with the aim of their mechanistic understanding. Thereby, the final hope is to reduce laboratory experimental efforts by correctly identifying mechanisms and nodes relevant for a certain process. Nevertheless, the more models grow in size, the more also computational efforts become demanding. In this context, we provide a new and crucial method to narrow down the complexity of *in silico* investigation by determining dynamic drivers which are sufficient to determine the whole phenotypical landscape. Given that we already show above that our set detects promising intervention and therapeutic targets, we now want to depict the overall process of moving from model establishment, to simulation, to identification of dynamic drivers, and finally to bench for *in vitro* validation.

**Fig. 3:**
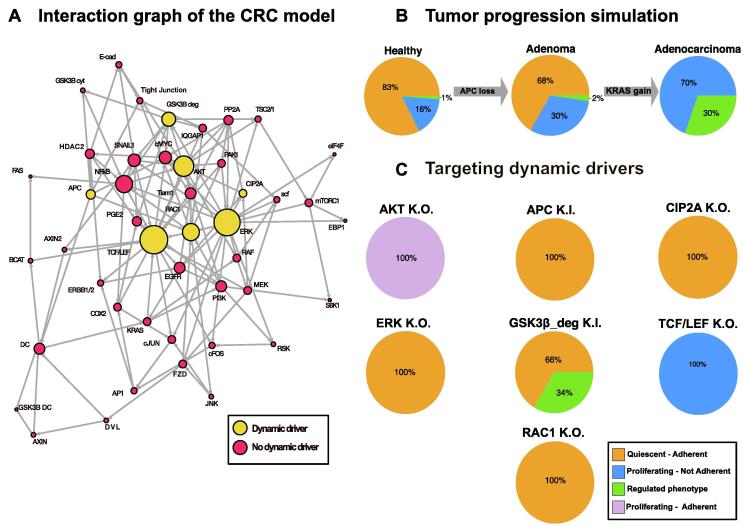
Modeling colorectal cancer progression and intervention. (A) An interaction graph of the colorectal cancer (CRC) model is shown. Dynamic drivers are highlighted in yellow. The size of the circles is proportional to the z-transformed connectivity of the node. (B) Phenotypical distribution during tumor progression is depicted by pie charts. (C) Phenotypical distribution after dynamic driver intervention. In general, phenotypes are assigned based on the activity of nodes responsible for proliferation and migration (see also Appendix Figs. A.2-A.3 and Appendix Method A.4). Please note that simulations were performed considering the opposite behavior of each dynamic driver compared to the adenocarcinoma state (e.g. AKT is active in the adenocarcinoma phenotype, therefore a knockout was performed). (For interpretation of the references to colour in this figure legend, the reader is referred to the web version of this article.)

To this purpose, we constructed a new literature-based Boolean network model (Table 2) dealing with the crosstalk of two frequently mutated pathways in colorectal cancer (CRC) – Wnt and MAPK [81] with the final aim of predicting new therapeutic targets. Within this scope, we focused on a severe phenotype of colorectal cancer with APC loss and mutated KRAS (adenocarcinoma state). The final model contains 45 nodes and 256 interactions (Fig. 3A) and is able to reproduce the progression of CRC (Fig. 3B, Fig. A2). After assessing that the model’s dynamic landscape represents cancer progression, we applied our new method to identify dynamic drivers. Thereby, we identified seven nodes responsible for the complete network dynamics (Fig. 3A).

**Table 2:** Boolean functions of the Wnt/MAPK network Interactions are described by logical connectives AND (&), OR. (|), and NOT (!). All proteins are abbreviated by the current nomenclature. A detailed biological description of the Boolean functions is provided on GitHub: https://github.com/sysbio-bioinf/DynamicDriverSets.

Node	Boolean function
EGFR	ERBB1/2 & PGE2 &!ERK
KRAS	EGFR &!DC
RAF	KRAS &!ERK &!AKT
MEK	RAF
scf	IQGAP1 & RAF & MEK
ERK	(scf | PAK1) &!PP2A
eIF4F	ERK | mTORC1
EBP1	!ERK &!mTORC1
MYC	(ERK | TCF/LEF) &!APC & (!PP2A | CIP2A) &!GSK3 β_deg_ & ERK
cJUN	(ERK | TCF/LEF | COX2) & JNK
PI3K	PGE2 | EGFR | KRAS
AKT	(PI3K | PAK1 | SNAIL1) &!PP2A & (NF-κ B | TCF/LEF | SNAIL1)
TSC1/2	GSK3 β_deg_ &!ERK &!AKT
mTORC1	!TSC1/2
S6K	mTORC1 & PI3K
TIAM1	(EGFR | AKT) &!PP2A & (MYC | TCF/LEF)
RAC1	(TIAM1 | IQGAP1 | mTORC1 | PI3K | FZD) &!APC
JNK	RAC1
PAK1	RAC1 &!PP2A
IQGAP1	!GSK3 β_deg_
PGE2	COX2 | (SNAIL1 & HDAC2)
HDAC2	!APC & MYC
ERBB1/2	HDAC2 | AP1 | TCF/LEF
cFOS	(TCF/LEF | ERK) & (ERK | RSK1/2)
RSK1/2	PI3K & ERK
AP1	cFOS & cJUN
COX2	AP1 | NF-κ B | TCF/LEF
FASR	NF-κ B &!CTNNB1
NF-κ B	(RAC1 | ERK | AKT) & HDAC2 & GSK3 β_cyt_
CDH1	(!SNAIL1 &!HDAC2 &!AKT) | (!SNAIL1 & HDAC2 & AKT) | (!SNAIL1 &!HDAC2 & AKT) | (!SNAIL1 & HDAC2 &!AKT) | (SNAIL1 &!HDAC2 &!AKT) | (SNAIL1 & HDAC2 &!AKT) |. (SNAIL1 &!HDAC2 & AKT)
Tight junctions	CDH1 & (!IQGAP1 | APC | (RAC1 & IQGAP1))
SNAIL1	((AXIN2 | ERK | NF-κ B) &! GSK3 β_deg_) | (AXIN2 & GSK3 β_deg_)
AXIN2	TCF/LEF
FZD	MEK | ERK | JNK
DVL	FZD
GSK3 β_deg_	!PGE2 &!AKT &!ERK &!NF-κ B
GSK3 β_cyt_	!APC | GSK3 β_deg_
GSK3 β_DC_	AXIN1
APC	APC
AXIN1	!DVL
DC	!DVL & GSK3 β_DC_ & APC & (AXIN1 | AXIN2)
CTNNB1	!DC
TCF/LEF	CTNNB1 & KRAS & RAC1 & (PAK1 | AKT | MEK | IQGAP1 | TIAM1 | NF-κ B | SNAIL1)
PP2A	!CIP2A
CIP2A	EGFR | MEK | ERK

Next, we analyzed the potential of the identified dynamic drivers as intervention targets for CRC. To do so, we performed *in silico* intervention simulations with these nodes based on a progressed cancer condition phenotype (loss of APC and active KRAS, adenocarcinoma phenotype) and compared the attractor landscape to the unperturbed network (Fig. 3C and Appendix Fig. A3, and Appendix Methods A.4). Thereby we focused on the potential of the interventions to inhibit proliferative and/or migratory traits (a detailed analysis of the attractor patterns is provided in Appendix Methods A.4). Additionally, we screened if drugs are available to potentially treat human beings (Table 3 and Appendix Methods A.4). This deep investigation indicated that the dynamic drivers ERK and CIP2A are the most promising unexplored intervention targets in the landscape of CRC. The rationale of the selection of dynamic drivers to further test is summarized in Table 3 and described in detail in the Appendix Methods A.4.

**Table 3:** Rationale of selection of dynamic drivers for further laboratory validation Please note that simulations were performed considering the opposite behavior of each dynamic driver compared to the adenocarcinoma state and the activity of cMYC and Tight Junctions nodes.

Dynamic Driver	*In silico* perturbation effectson the adenocarcinoma phenotype (see also Fig. 3C, Appendix Fig. A.3 and Appendix Method A.4)		Available small molecules (See also Appendix Method A.4)	Known for resistance/inefficacy in patients (See also Appendix Method A.4)	Target approaches in humans (See also Appendix Method A.4)	Targeted in CRC KRAS patients (See also Appendix Method A.4)
	Proliferation	Adhesion				
AKT	AKT knockout does not change proliferative potential	AKT knockout restores adhesion	[85]	[86]	[85]	NCT 01,333,475 [87]; NCT01802320 [88]
APC	APC knock-in inhibits proliferation	APC knock-in restores adhesion	[89]	–	–	–
CIP2A	CIP2A knockout inhibits proliferation	CIP2A knockout restores adhesion	[83], [90], [91]	–	[92] (As derivative of Erlotinib)	–
ERK	ERK knockout inhibits proliferation	ERK knockout restores adhesion	[82], [93], [94]	–	[94], NCT03417739, NCT02994732, NCT02296242, NCT01781429, NCT03454035, NCT03698994, NCT02608229, NCT02465060, NCT03155620	–
GSK3β	GSK3β knock-in inhibits proliferation	GSK3β knock-in restores adhesion	*In vitro* shown mechanisms [95], but no small molecules	–	–	–
TCF/LEF	TCF/LEF knockout does not change proliferation	TCF/LEF knockout does not affect adhesion	Problems of complex selectivity [96]	Specific inhibition of Wnt will destroy tissue homeostasis [97]. Need for cancer specific signals	–	–
RAC1	RAC1 knockout inhibits proliferation	RAC1 knockout restores adhesion	Developing selective inhibitors is still an open issue [98]	–	–	–

To test now the power of the identified dynamic drivers as intervention targets, we treated the CRC cell line SW480 with the specific ERK inhibitor BVD-523 [82] and the specific CIP2A inhibitor TD-52 [83]. Coupling kinase and phosphatase inhibitors has been applied to prevent known insurgence of resistance of MEK and RAF inhibitors [84]. Even if this is not reported in the case of ERK and no resistance mechanisms are known yet from clinical setups (Appendix Table A.1), we tested the combination of the two targets. This follows the hypothesis that also ERK inhibitors might be enforced by further phosphates inhibition.

**Fig. 4:**
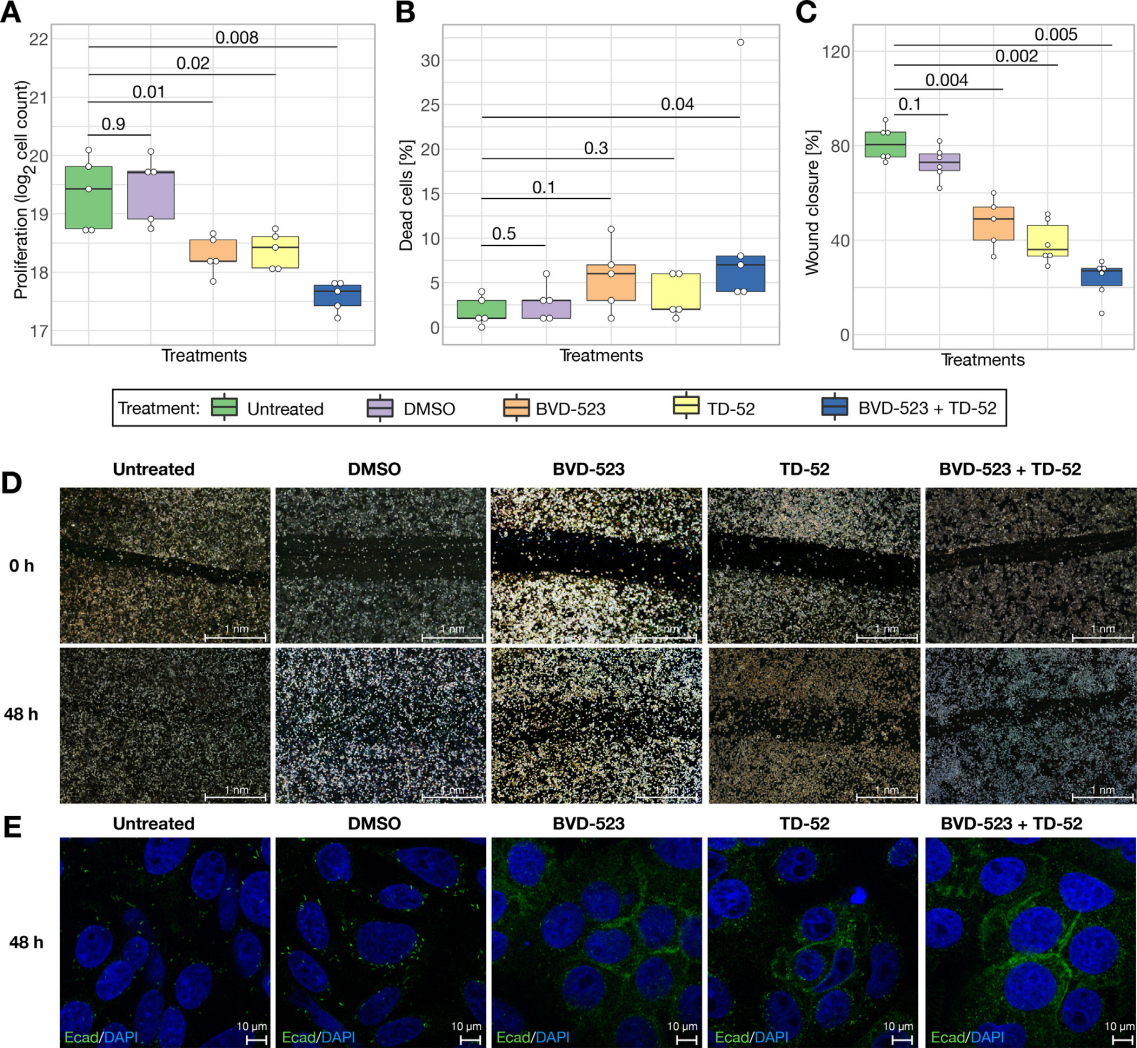
Targeting dynamic drivers *in vitro*. ERK and CIP2A identified as dynamic drivers were targeted individually and in combination. (A) Cell counts from proliferation assay after 24 h post-treatment (n = 5, Wilcoxon test). For both of the single drug treatments, a 2-fold reduction was detected. The combined treatment led to a 3.4-fold decrease. (B) The percentage of dead cells from the proliferation assay shows no significant differences in apoptosis (n = 5, Wilcoxon test). (C, D) The percentage of wound closure after 48 h post-treatment indicates a reduced migratory potential (BVD-523: n = 5, otherwise: n = 6, Wilcoxon test). (E) Merged confocal microscope pictures of E-cadherin staining (green) and colored nuclei (blue) 48 h post-treatment. Treatment of dynamic drivers restored E-cadherin at the cell membrane. We adjust p-values via Bonferroni corrections and assume significant results if p < 0.05. p-values are depicted on top of each comparison bar. (For interpretation of the references to colour in this figure legend, the reader is referred to the web version of this article.)

SW480 cells are known to have a loss of function alterations of APC. Since this loss of APC is associated with increased proliferation [99] and migration [100] as well as loss of cell adhesion [101], we studied the impact of our interventions on these effects. Treatment with either BVD-523 or with TD-52 reduced the proliferative potential of SW480 by 2-fold (mean values, Fig. 4A) within 24 h in comparison to untreated or DMSO treated controls without increasing apoptosis (Fig. 4B). By combining both approaches of inhibition, an even stronger mean inhibitory effect of 3.4-fold was achieved (Fig. 4A).

Moreover, the migratory potential of ERK or CIP2A treated SW480 cells was significantly reduced (Fig. 4C-D). Depth-characterization of the *in silico* interventions of the dynamic drivers ERK and CIP2A indicated a restoration of E-cadherin at the cell membrane (Appendix Fig. A.4). This might explain the reduced migratory potential observed after inhibiting ERK or CIP2A. Staining of E-cadherin could support this assumption (Fig. 4E, Appendix Fig. A.4).

Our workflow could successfully show how the investigation of dynamic drivers can be implemented to guide *in vitro* validation of new intervention targets in a certain tumor landscape. Here, we could show that the dynamic driver set helps to quickly restrict candidates for intervention, especially in large networks. Besides, predictions on the perturbation of candidate targets were confirmed in our *in vitro* experiments. Altogether, we presume that detecting these dynamic drivers sets can be helpful and supportive in translating large *in silico* setups into *in vitro* validation. Notably, 90 *in silico* perturbation experiments should have been performed and singularly evaluated without the dynamic driver set to narrow down the intervention candidates.

## 4 Discussion

In the present work, we set up an approach to identify sets of dynamic drivers responsible for determining the entire dynamic behavior of the system. Our approach is developed in the context of Boolean network models. Using dynamic models requires collecting and integrating existing knowledge, which can be time-consuming. Next, mathematical terms need to be derived to model regulatory interactions between the models’ compounds. This modeling process might require extensive literature and data research. However, Boolean networks as models have the great advantage of allowing dynamic simulations of large networks by not requiring the knowledge of precise kinetic parameters.

Consequently, less data is needed than in other dynamic models such as systems of differential equations. These parameters are often unknown, and their automatic inference would require significant experimental efforts, especially in modeling extensive pathway crosstalk. In addition, the latest modeling approaches in the context of Boolean networks showed the potentiality to perform attractor searches up to 100,000 nodes [102], bringing the dynamic investigation to genome-size networks. From a different perspective, Boolean models might be considered an oversimplification of the actual complexity of biological systems. Nevertheless, other previous research efforts have shown that predicting phenotypes via Boolean modeling is a winning strategy, further sustained by experimental validation on model-based predictions [9], [10], [103], [104].

Controlling biomolecular networks has become a demanding task considering shifting the phenotypical landscape towards the desired behavior. For this reason, different studies proposed methods to identify dynamic driver sets able to control single phenotypes based on logic gene regulatory networks [5], [32], [35], [36]. From this perspective, different approaches are possible. Some works focus on driving single starting states toward the desired phenotype [13], [105], [106]. Others, instead, investigated how to drive any possible starting state to a single desired phenotype [5], [107], [108], [109].

Nevertheless, methods to identify control sets that define the complete phenotypical landscape are still missing. In this direction, Choo et al. [32] proposed a method to drive any possible starting state towards a set of phenotypes sharing the same sink (phenotypical node, e.g., “apoptosis”). While this work targets multiple phenotypes, it relies on nodes that are not commonly present in all logical biomolecular networks.

In addition, the diversity of the phenotypical landscape is still limited. These methods have in common their requirement of precise knowledge of the desired phenotype to be targeted. Yang and colleagues [110], instead, by defining the concept of Logical Domain of Influence (LDOI) of a particular node state, were able to uncouple the identification of intervention targets to the attractor search. By studying the properties of Boolean operators, they identified by starting from a fixed node state the sets of nodes whose activities are determined by the applied perturbation (the LDOI of the fixed node). Again, while this method does not require an exploration of the state space of the examined Boolean network, it is still restricted to controlling only a subset of activities related to a specific target phenotype.

Here, instead, we approached the control problem from a different perspective. We set up a method to identify driver sets that alone can determine the whole attractor landscape of the system. Our approach is independent of the precise knowledge of the phenotypical landscape, and we could show that our identified dynamic drivers can be targeted independently from each other. Interestingly, this “unbiased” exploration or prediction of dynamic landscapes has been applied mostly via topological-based methods [111], [112]. Nevertheless, our method strongly relies on the presence of a Boolean model. In contrast, to, e.g., hubs as potential targets, which only need directed graphs, our approach uses this additional information to make a tailored prediction of dynamic influencing nodes.

As a further example of this, Weidner et al. [112] identified a set of topological measures able to capture the dynamic properties of Boolean networks. While such methods are fast and scale up well with the increase in network sizes, they still retrieve larger sets of nodes than dynamic-based ones. In the case of Weidner et al. [112], the intersection of the two selected topological-based measures led to a reduction of nodes of interest of around 30%. This selection was, in that case, further reduced by focusing on subsets of interest-based on other properties such as connectivity.

We applied our method to 35 previously published Boolean networks (Table 1). Similar to previous studies [5], [32] tackling single phenotypical control, we could show that a small set of nodes also determine the whole phenotypical landscape independently of the network size. By comparing our sets to previously suggested topological and dynamic measures [71], we showed that our sets of dynamic drivers are identifiable only via our approach, with reduced overlap with previously established methods. Interestingly, our method combines nodes previously indicated as important for the network dynamics [5], [67], [68], [71], [113] and couples them with new ones. Highly connected components represent a striking example of this. Our results suggest that highly connected nodes are not the only relevant components defining our driver sets. In accordance, Liu and colleagues [13] previously indicated that driver sets tend to avoid hub nodes. This might appear to contrast the common assumption that highly connected nodes are master regulators of biological processes [114]. However, our results indicate that both theories can co-exist by showing that hubs are a subset of dynamic driver genes. Additionally, both our *in silico* and *in vitro* case studies showed that the effect of targeting our dynamic drivers is independent of their degree of connectivity. Moreover, we highlighted a promising role of coupling individual dynamic drivers as intervention targets.

We envision that our approach can be applied to ease up the transition between *in silico* prediction and experimental setup. For this reason, we established a workflow in the context of new therapeutic targets for CRC. Starting from a large model, we could reduce the search of potential targets to only seven nodes, all able to affect network dynamics. Interestingly, while our model is shown to be enriched for drug targets involved in clinical trial use, the set of dynamic drivers tends to exclude targets known to raise resistance to treatment in cancer patients (Appendix Fig. A.5, and Appendix Table A1). Since resistance mostly arises from reactivation mechanisms that limit the dynamic impact of the intervention, we conclude that evaluating the driver sets on a dynamic level helps avoid the selection of targets inducing these resistance mechanisms. In addition, while four out of seven of our dynamic drivers have reported drugs in clinical trials, the ones not yet in clinical use might still be interesting as new future targeted interventions.

On these grounds, we deepened our analysis on the driver set by coupling our *in silico* prediction with knowledge of drug and potential clinical applications; we could select two previously uninvestigated therapeutic targets in CRC and successfully test them *in vitro*. Our results highlight the advantages of our method: 1.) Our sets of dynamic drivers are independent of the precise phenotype context. In the CRC scenario, this translates into their applicability to different cancer stages or their role as disease drivers. A striking example of this is APC, both a disease driver and a potential therapeutic target. 2.) Our method efficiently scales down the computational effort. Considering that the established model consists of 45 nodes, 90 *in silico* knock-in and knockouts should be simulated and evaluated to determine promising single targets. This number triples if one is interested in the applicability to different cancer stages and disease drivers and exponentially scales up by target combinations. 3.) Dynamic drivers can be targeted independently from each other, leaving a wide range of possibilities for both single and combinations of interventions.

In the present work, we designed an approach to identify dynamic drivers able to control the whole phenotypical landscape of a biomolecular network. To the best of our knowledge, ours is the first study to address this specific question. Our results support the understanding of characteristics governing network dynamics and can be promising in guiding drug target identification.

## 5 Conclusion

We presented a computational approach to retrieve minimal sets of dynamic driver nodes whose activities are responsible for the entire attractor landscape of the simulated system. We could study both the topological and dynamic features of our retrieved sets of dynamic drivers by applying our approach to a wide range of biologically motivated networks. Our results indicate that dynamic driver nodes are less highly connected than hub nodes, and their perturbation leads to relevant shifts in the dynamics of the analyzed networks. We could associate loss of dynamic drivers with disease drivers or therapeutical interventions. Finally, we could show their application as a therapeutic intervention in a case study where we presented a new dynamic model to study colorectal cancer progression.

## Declaration of Competing Interest

The authors declare that they have no known competing financial interests or personal relationships that could have appeared to influence the work reported in this paper.
