# Supplementary material for: Identification of dynamic driver sets controlling phenotypical landscapes
Source: Comput Struct Biotechnol J. 2022 Apr 2;20:1603–17. doi: 10.1016/j.csbj.2022.03.034 (PMC9010550; doi:10.1016/j.csbj.2022.03.034)
Supplement: Supplementary data 1 [file mmc1.docx]

**Identification of dynamic driver sets controlling phenotypical landscapes**

Silke D. Werle^1,4^, Nensi Ikonomi^1,4^, Julian D. Schwab^1,4^, Johann M. Kraus^1^, Felix M. Weidner^1^, K. Lenhard Rudolph^2^, Astrid S. Pfister^3^, Rainer Schuler^1,5^, Michael Kühl^3,5^, Hans A. Kestler^1,5,*^

^1^Institute of Medical Systems Biology, Ulm University, 89081 Ulm, Baden-Wuerttemberg, Germany

^2^Leibniz Institute of Aging – Fritz Lipman Institute, 07745 Jena, Thuringia, Germany

^3^Institute of Biochemistry and Molecular Biology, Ulm University, 89081 Ulm, Baden-Wuerttemberg, Germany

^4^These authors contributed equally to this work

^5^These authors also contributed equally to this work

^*^Correspondence: hans.kestler@uni-ulm.de

**Figures**

**Figure A.1:** Dynamic driver nodes characterization. (A) Altered network diameter after perturbation. The effect is shown as an absolute value in terms of network diameter change after single perturbations of dynamic drivers, hubs, or other nodes. (B) Functions canalyzed by a single node in the set of dynamic drivers, hubs, and other nodes. Wilcoxon test was performed with Bonferroni correction. p-value < 0.05 is considered significant.

**Figure A.2:** Simulation of progression of colorectal cancer. The detailed attractor pattern of Figure 3B is shown here. The attractors obtained for the unperturbed network (healthy), APC loss (adenoma), and APC loss, and KRAS gain of function (adenocarcinoma) are represented below each condition. Below each attractor, the corresponding basin size is reported as the percentage of the whole state space. Network components are listed on the left and grouped by signaling cascades. The activity state of each component is displayed by colored rectangles.

**Figure A.3:** Intervention screening with implicants. Detailed attractor pattern of Figure 4C. Identified dynamic drivers were knocked-out (ko) or overexpressed (ki) in-silico and the impact of this intervention was analyzed based on the obtained attractors. Simulations were performed with inactive APC (fixed to 0) and constantly active KRAS (fixed to 1). Network components are listed on the left and grouped by signaling cascades. The activity state of each component is displayed by colored rectangles.


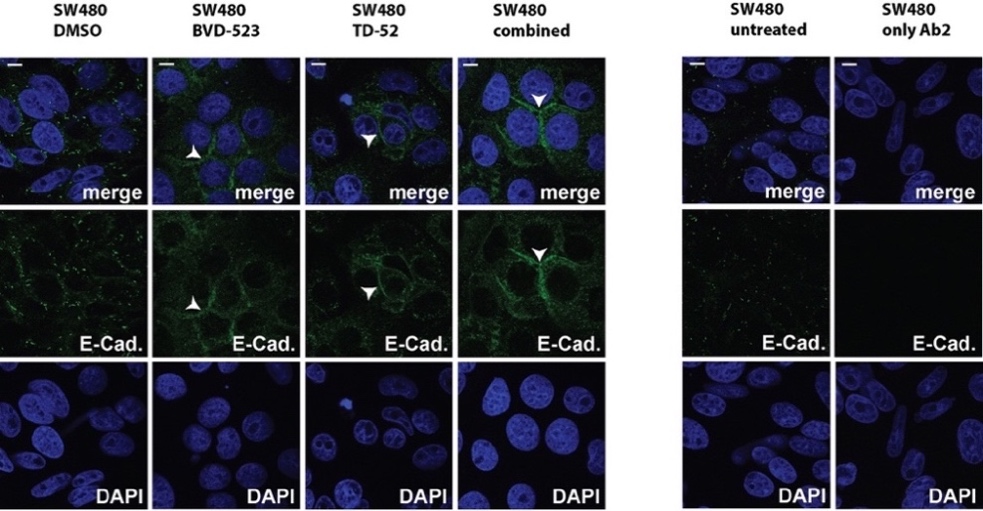


**Figure A.4:** E-cadherin staining of SW480 cells. Representative confocal images of SW480 cells stained with E-cadherin (E-Cad) antibody and for DAPI are depicted. Single treatments show an increased E-cadherin signal and a localization at the cell membrane when compared to controls. Combined treatment shows an enhanced signal localized at the cell membrane. Treatment and controls (DMSO and untreated) are indicated above the figure. The first row shows merged channels (E-Cad and DAPI) for each setup as indicated. A negative control is shown in the last column using only the secondary antibody.

**Figure A.5:** Euler diagram showing the results of Table A.1. Intersections of dynamic drivers, drug targets reaching clinical trials, and targets who have shown resistance to treatment in cancer patients are represented. The rest of the nodes of the CRC model not included in any of these subgroups are indicated as “other nodes”.

**Tables**

**Table A.1:** Analysis of the distribution of drug targets in the CRC model. The availability of specific drugs currently in the clinical trial phase for cancer therapy (at least one) was evaluated via clinicaltrails.gov and the therapeutic target database. In addition, a literature search was performed to search for those targets who have also reported resistance in clinical settings. The dynamic drivers are highlighted in bold. A checkmark indicates a positive hit, while “-“ indicate a missed hit.

| **Node** | **Drug target in clinical Trial** | **Clinical Trial Reference** | **Reported Resistance in Clinical evaluation** |
| --- | --- | --- | --- |
| EGFR | 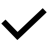 | NCT03122717, NCT03392246,  NCT01750918,  NCT00243854,  NCT03705507,  (…) | [1–3] |
| KRAS | 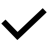 | NCT01199263,  NCT00655161,  NCT00531401,  NCT01322815,  NCT00655161,  NCT00300950,  (…) | [4–8] |
| RAF | 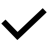 | NCT00492986,  NCT01877811,  NCT04190628,  NCT01225536,  NCT01726738,  (…) | [9–11] |
| MEK | 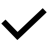 | NCT04615312,  NCT02094872,  NCT01726738,  NCT05080361,  NCT02049801,  NCT04216953  NCT04967079,  (…) | [12–14] |
| **ERK** | 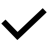 | NCT03417739,  NCT04488003,  NCT04145297,  NCT05221320,  NCT02296242,  NCT01781429,  (…) | Not available yet from clinical results, hypothesized potential resistance [15, 16]. |
| eIF4F | 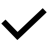 | NCT01675128,  NCT00903708,  NCT01234038,  NCT01234025 | [17, 18] |
| 4EBP1 | - | - | - |
| cMYC | 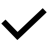 | NCT02314052,  NCT02110563 | - |
| cJUN | - | - | - |
| PI3K | 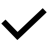 | NCT01923168,  NCT04342117,  NCT01816984,  NCT01791478,  NCT01629615,  NCT04330625,  (…) | [19, 20] |
| **AKT** | 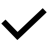 | NCT01802320, NCT02177682,  NCT01907815,  NCT01349933,  NCT01253447,  NCT01277757,  NCT01333475,  NCT01090960,  NCT01915576,  (…) | Predictive markers for response and resistance to AKT inhibitors are still an unmet need. Some markers have been suggested in pre-clinical contexts. One patient case study has suggested a resistance mechanism to AKT inhibitors. [21–24] |
| TSC1/2 | - | - | - |
| mTORC1 | 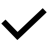 | NCT01545817,  NCT00863655,  NCT00510068,  NCT01351350,  NCT01420081,  NCT02305810,  NCT01628913,  (…) | [25–27] |
| S6K1 | - | - | - |
| Tiam1 | - | - | - |
| **RAC1** | - | - | - |
| JNK | - | - | - |
| PAK1 | - | - | - |
| IQGAP1 | - | - | - |
| PGE2 | - | - | - |
| HDAC2 | 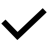 | NCT02836548,  NCT04512534,  NCT02569320,  NCT01075308,  NCT00697879,  (…) | [28–30] |
| cFOS | - | - | - |
| RSK | 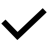 | NCT04115306 | - |
| AP1 | - | - | - |
| COX2 | 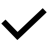 | NCT00046839,  NCT02429427,  NCT03864575,  (…)  Generally proposed as combination or coadiuvant therapy | - |
| FAS | 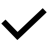 | NCT00559117,  NCT04166383,  NCT03398655,  NCT01229865,  NCT04406272,  (…) | - |
| NF-kB | - | - | - |
| CDH1 | - | - | - |
| SNAIL1 | - | - | - |
| AXIN2 | - | - | - |
| FZD | 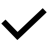 | NCT01345201,  NCT02005315,  NCT01957007,  NCT01973309 | - |
| DVL | - | - | - |
| **GSK3B** | 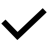 | NCT01214603,  NCT01287520,  NCT01632306,  (…) | - |
| **APC** | 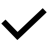 | NCT04454151,  NCT02354560 | - |
| AXIN1 | - | - | - |
| CTNNB1 | - | - | - |
| **TCF/LEF** | - | - | - |
| PP2A | 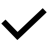 | NCT03027388,  NCT01837667,  NCT04560972 | - |
| **CIP2A** | - | - | - |

**Methods**

**Method A.1:** Pseudocode for the heuristic approach.

The heuristic is defined in terms of significance. Here, the significance of a node is maximal if its transition function depends on its value. Otherwise, the significance of node g is equal to the number of nodes whose transition function depends on g. Therefore, in each iteration, the heuristic selects a compound g with the highest significance until a set of implicant nodes is found.

**input** : Boolean network $F=(F_{1},..., F_{n})$

**output** : A minimal implicant set

1 $G\leftarrow\emptyset$

2 **repeat**

3 $w\leftarrow nodeWeight(F,G)$

4 $g\leftarrow x_{i}, where w(x_{i})=max(w)$

5 $G\leftarrow G\cup\{g\}$

6 $observables\leftarrow observableStates(F,G)$

7 **until** observables;

8 **return** G

9 $nodeWeight(F,G)$

10 $\mathbf{for} all nodes x_{i} in G \boldsymbol{do}$

11 $remove rule F_{i} and all occurence of x_{i} from F$

12 $\mathbf{end}$

13 $\mathbf{for} all nodes x_{i}, where F_{i} depends only on x_{i} \mathbf{do}$

14 $w(x_{i})\leftarrow n$ *// max weight*

15 $\mathbf{end}$

16 $\mathbf{for} all nodes x_{i}, where F_{i} depends only on x_{j}, i\neq j \mathbf{do}$

17 $w(x_{i})\leftarrow0$ *// min weight*

18 $\mathbf{end}$

19 $\mathbf{for} all nodes x_{i}, where {w(x}_{i}) is not already set \mathbf{do}$

20 $w(x_{i})\leftarrow the number of j such that F_{j} depends on x_{i}$

21 $\mathbf{end}$

22 **return** w

**Method A.2:** Pseudocode for the exhaustive approach.

As a reference for the heuristic search, we implemented an exhaustive search algorithm.

**input** : Boolean network $F=(F_{1},..., F_{n})$

**output** : Minimal implicant set

1 $success\leftarrow false$

2 $implicants\leftarrow\emptyset$

3 $\mathbf{for} increasing values of k \mathbf{do}$

4 $\mathbf{for} all possible selections G=\{x_{i_{1}}...,x_{i_{k}}\} of k nodes \mathbf{do}$

5 $observables\leftarrow observableStates(F,G)$

6 $\mathbf{if} observables \mathbf{then}$

7 $success\leftarrow true$

8 $implicants\leftarrow implicants\cup\{G\}$

9 $\mathbf{end}$

10 $\mathbf{end}$

11 $\mathbf{if}\boldsymbol{success}\mathbf{then}$

12 $\mathbf{return implicants}$

13 $\mathbf{end}$

14 $\mathbf{end}$

15 $observableStates(F,G)$

16 $\mathbf{for} every assignment a to the k nodes of G \mathbf{do}$

17 $let the assignment a be undefined for all remaining n-k nodes$

18 $evaluate F until an attractor A_{a} is found$ *// refinement*

19 $\mathbf{if} A_{a} is undefined on some node \mathbf{then}$

20 $\mathbf{return}$ false

21 $\mathbf{end}$

22 $\mathbf{end}$

23 $\mathbf{return true}$

**Method A.3:** Pseudocode for the network reduction.

Network reduction was performed to reduce the search space and accelerate the analysis for large Boolean network models. This was achieved by removing nodes that do not regulate other nodes. The procedure was repeated until all superfluous nodes were removed. The code will yield the Boolean functions of a reduced network.

**input** : Boolean network $F=(F_{1},..., F_{n})$

**output** : Reduced network

1 $In\leftarrow\{x_{i} | F_{i}(x)=x_{i}\}$

2 $\mathbf{while} \exists x_{l}\in F,x_{i_{l}},...,x_{i_{k}}\in IN : F_{l}(x)=f(x_{i_{l}},...,x_{i_{k}}) \mathbf{do}$

3 $remove F_{l} from ruleset F$

4 $substitute all occurrences of x_{l} in F by f(x_{i_{l}},...,x_{i_{k}})$

5 $\mathbf{end}$

6 $\mathbf{while} \exists x_{l}\in F : x_{l} is not contained in any rule F_{i} \mathbf{do}$

7 $remove F_{l} from ruleset F$

8 $\mathbf{end}$

**Method A.4:** CRC model construction strategy and dynamic analyses.

*Model setup rationale*

Colorectal cancer (CRC) is the third most common cancer in men and women and accounts for approximately 10% of cancer-related death in developed countries [31]. Initially, CRC arises from a polyp that evolves into an adenoma and further into an adenocarcinoma, which can then infiltrate tissues gaining in invasiveness, stem-like behavior and proliferation potential [31]. In this picture, Wingless (Wnt) and mitogen-activated protein kinase (MAPK) signaling play a main role in synergistically inducing colon carcinomas. The progression of CRC takes place in a multi-step manner and is driven by a stepwise accumulation of mutations [32–34]. In particular, adenomatous polyposis coli (APC) loss of function mutations are present in 90% of CRC and are typical of early stages of CRC [35]. On the other hand, aberrant activation of RAS through mutation (KRAS), is considered a successive temporal step, occurring in up to 50% of CRC patients [36]. Interestingly, these two mutations alone are not sufficient to lead to severe cancer phenotypes (such as in familial adenomatous polyposis (FAP) patients with loss of APC). Whereas, when present together they lead to a severe cancer condition with marked invasive and proliferative traits [31, 32, 34, 37, 38]. Considering the relevance of the crosstalk in CRC progression and the fact that its dynamics are still poorly understood [36, 37, 39], we propose here a Boolean network model depicting the main elements.

*Interpretation of the CRC network dynamic behaviour*

In constructing our network, we considered relevant components of both Wnt and MAPK cascade and took into account different levels of regulation (from gene expression to protein-protein interactions). In order to reconstruct the stepwise accumulation of mutations characterizing CRC, we studied the dynamic evolution of our model in three different starting conditions: unperturbed (healthy phenotype), APC loss (APC=0), and a further acquisition of KRAS (KRAS=1). Resulting attractors and basins of attractions were evaluated to access the capability of the crosstalk model to resemble CRC progression (Figure A.2). When the network is simulated in unperturbed conditions (any node is fixed at the beginning of simulations), we obtain a set of attractors resembling a healthy condition. Most of our attractors resemble a quiescent condition. Nevertheless, a subpopulation of starting states leads to a proliferative-like attractor. This is also expectable since, in the intestinal crypts, a high level of proliferation is required for the turnover of the tissue itself. In particular, elevated Wnt signaling are present at the bottom of the crypts [40]. The first step in cancer progression is the loss of function of APC. Our *in silico* simulation with APC loss revealed attractors that represent either accumulation of β-catenin (CTNNB1) or further activation of RAS signaling. Thus, they suggest the presence of a different adenoma condition where the majority of cells (larger basin of attraction) are aberrantly accumulating CTNNB1 without triggering proliferation. On the other hand, a subset of the total population is showing activation of RAS signaling, sustaining a more severe phenotype. Our results support the idea that only APC loss followed by triggering of Wnt signaling is not enough to induce a severe cancer condition. However, it can still sustain a feedback activation of RAS signaling, promoting cancer-genesis [36, 37]. The missing of nuclear accumulation of CTNNB1 has also been observed in adenoma patients, supporting our results [41, 42] (Appendix Figure A.2). Moreover, the shift of the basin of attraction towards a single state proliferative attractor from healthy to APC loss simulations supports the hypothesis that the adenoma state is associated with an expansion of proliferative compartments of intestinal crypts [43, 44] (Appendix Figure A.2). In order to simulate progression of disease to a more severe phenotype, we further considered the introduction of activating RAS mutation (KRAS) in our model in addition to the loss of APC. The results of our simulations show a set of two attractors both depicting high activation of Wnt and MAPK and crosstalk regulated components (Figure A.2). In any case, one attractor shows a milder phenotype with three cycling states (defined as regulated phenotype in Figure 3 of the main). This is due to residual activity of protein phosphatase 2A (PP2A), known to regulate the degradation of different pro-oncogenic components [45, 46]. Indeed, PP2A downregulation is a common event in CRC, and patients with residual activity of the phosphates show a better prognosis and overall survival [45, 46]. These initial simulations showed that our newly established model is able to reproduce dynamic behavior known from CRC initiation and progression. Thus, we applied it to search for promising intervention targets based on dynamic characteristics.

*Attractor evaluation of dynamic drivers*

Within our newly established CRC network, we identified seven dynamic drivers (TCF/LEF, ERK, APC, CIP2A, AKT, RAC1, ${GSK3\beta}_{degratation}$). As a second step, we investigated the effect on the attractor landscape of biologically meaningful intervention (e.g. knockout or overexpression) for each individuated dynamic driver in order to evaluate possible therapeutic interventions in CRC (Figure A.3).

Proliferation was evaluated via the activity of cMYC nodes, and adhesion via the activity of the “Tight Junctions” node. Hence, we would consider the attractor pattern to improve when cMYC and Tight Junctions would get reactivated after perturbation. Herewith, we briefly report and comment our results for the perturbation of each suggested dynamic drivers together with the rational of selection of ERK and CIP2A for *in vitro* validation.

**TCF/LEF:** The knock-out of T-cell factor/lymphoid enhancing factor (TCF/LEF) does not heavily affect the final attractors in terms of proliferation and adhesion, except for the loss of a cyclic attractor. This can be explained from the fact that extracellular-signal regulated kinase (ERK) has a redundant effect on downstream targets of TCF/LEF in the crosstalk [36, 47]. In addition, the design of small molecules for this target is still challenging due to issues in the complex selectivity and alteration of tissue homeostasis [48]. Hence, this target was excluded for further validation.

**ERK:** After ERK knock-out only one attractor is found that shows an inhibition of proliferation and invasion potential (inhibition of cMYC and reactivation of Tight Junctions). The presence of ERK targeting small molecules and first encouraging clinical trials results for solid tumor patients further encourage the selection of this target [49, 50]. MAPK (EGFR, RAS, RAF, and MEK) inhibitors are well known to induce resistance in patients (See Appendix Table A.1). All resistance mechanisms to these inhibitors go through the reactivation of ERK, which is the most distal kinase of the cascade. Hence, ERK inhibitors are considered particularly promising since they may provide the opportunity to avoid or overcome the resistance from targeting upstream compounds of the cascade [50]. This is supported by preclinical evidence that inhibition of ERK by small-molecule inhibitors acted to both inhibit the emergence of resistance and overcome acquired resistance to BRAF and MEK inhibitors [49, 51–55]. While resistance is not yet observed in patients for ERK inhibitors, some other authors have also hypothesized potential resistance to ERK inhibitors in *in vitro* setups [15, 16]. Altogether, given the encouraging results on ERK inhibition from both our *in silico* simulations and available information on its targetability, we selected ERK as a target to be further validated.

**CIP2A:** cancerous inhibitor of PP2A (CIP2A) knockout causes a complete shift of the basin of attraction towards not proliferative and not invading phenotypes. Moreover, the presence of a small molecule selective for CIP2A (TD-52) derived from an already approved small molecule for lung tumor treatment further encourages the selection of this target [56, 57]. More in general, TD-52 is a quinzoline derivative, which is a very successful class of compounds with many clinically approved targets for cancer therapy [58, 59].

**AKT:** Protein kinase B (AKT) knockout causes only the inhibition of invasion potential, but not proliferation in the attractor landscape. In accordance, some AKT inhibitors have already been shown to produce a lack of response in CRC patients, discouraging the further selection of this target [60].

**RAC1:** Ras-related C3 botulinum toxin substrate 1 (RAC1) knockout has a huge impact on the attractor landscape, inhibiting both proliferation and invasion. In fact, only one single stage attractor is reached showing inhibition of most nodes in the network. This is probably connected to the fact that RAC1 is required for the shuttling of CTNNB1 in the nucleus [61]. However, such a huge impact on the phenotype could be undesired since it may also strongly affect healthy cells causing toxic effects. Moreover, developing RAC1 selective inhibitors is still an open issue, preventing the testing of this target for patients [62].

$\boldsymbol{GSK3\beta}_{\boldsymbol{degradation}}\boldsymbol{:}$ The ${GSK3\beta}_{degradation}$ node captures the degradation activity of ${GSK3\beta}$ connected to the phosphorylation and priming for degradation of its downstream targets [63]. Reactivation of ${GSK3\beta}_{degradation}$ degradation activity causes inhibition of proliferation and invasion. In addition, also two cycling attractors are reported, with more regulated activities. However, the expression of ${GSK3\beta}$ is also connected to pro-oncogenic activity due to potential different expression-related functions (represented in the model as other ${GSK3\beta}$ functional nodes) [63–65]. For this reason, some $GSK3\beta$ inhibitors have also reached clinical application (Appendix Table A.1). In the case of our targeting (knock in), the possibility of coexistence of these two functions relies on the presence of further proteins able to shelter the access of the kinase to the proteasome (an example is $\beta$ casein (CSN2)) [66]. Based on this, selective inhibitors of these sheltering proteins could be investigated to restore GSK3$\beta$ activity.

**APC:** APC loss of function mutations are present in 90% of CRC patients and connected to the first steps of tumor genesis [67]. Since any possible intervention based on regulatory interactions is feasible to target APC, we did not further investigate its possible reactivation (APC is an input node in the model). However, note that pre-clinical studies show promising results with a small molecule that induces synthetic lethality in APC truncated cells [68]. Interestingly, for adenoma patients, therapeutic approaches aimed to restore the expression of full-length APC are currently in the clinical trial phase (Appendix Table A.1).

Altogether, based on the attractor landscapes, the availability of small molecules, and the initial clinical and preclinical results, we selected ERK and CIP2A as therapeutic targets to be validated in wet laboratory experiments.

**References**

1. Huang WC, Yadav VK, Cheng WH et al. (2021) The MEK/ERK/miR-21 Signaling Is Critical in Osimertinib Resistance in EGFR-Mutant Non-Small Cell Lung Cancer Cells. Cancers (Basel). 13

2. Nagano T, Tachihara M, Nishimura Y (2018) Mechanism of Resistance to Epidermal Growth Factor Receptor-Tyrosine Kinase Inhibitors and a Potential Treatment Strategy. Cells. 7

3. Wu SG, Shih JY (2018) Management of acquired resistance to EGFR TKI-targeted therapy in advanced non-small cell lung cancer. Mol Cancer. 17:38.

4. Awad MM, Liu S, Rybkin II et al. (2021) Acquired Resistance to KRAS^G12C^ Inhibition in Cancer. N Engl J Med. 384:2382-2393.

5. Blaquier JB, Cardona AF, Recondo G (2021) Resistance to KRAS^G12C^ Inhibitors in Non-Small Cell Lung Cancer. Front Oncol. 11:787585.

6. Han Z, Zhou D, Wang J, Jiang B, Liu X (2022) Reflections on drug resistance to KRAS^G12C^ inhibitors and gene silencing/editing tools for targeting mutant KRAS in cancer treatment. Biochim Biophys Acta Rev Cancer. 1877:188677.

7. Riely GJ, Johnson ML, Medina C et al. (2011) A phase II trial of Salirasib in patients with lung adenocarcinomas with KRAS mutations. J Thorac Oncol. 6:1435-1437.

8. Zhao Y, Murciano-Goroff YR, Xue JY et al. (2021) Diverse alterations associated with resistance to KRAS(G12C) inhibition. Nature. 599:679-683.

9. Haarberg HE, Smalley KS (2014) Resistance to Raf inhibition in cancer. Drug Discov Today Technol. 11:27-32.

10. Lito P, Rosen N, Solit DB (2013) Tumor adaptation and resistance to RAF inhibitors. Nat Med. 19:1401-1409.

11. Luebker SA, Koepsell SA (2019) Diverse Mechanisms of BRAF Inhibitor Resistance in Melanoma Identified in Clinical and Preclinical Studies. Front Oncol. 9:268.

12. Brighton HE, Angus SP, Bo T et al. (2018) New Mechanisms of Resistance to MEK Inhibitors in Melanoma Revealed by Intravital Imaging. Cancer Res. 78:542-557.

13. Kun E, Tsang YTM, Ng CW, Gershenson DM, Wong KK (2021) MEK inhibitor resistance mechanisms and recent developments in combination trials. Cancer Treat Rev. 92:102137.

14. Tripathi R, Liu Z, Jain A et al. (2020) Combating acquired resistance to MAPK inhibitors in melanoma by targeting Abl1/2-mediated reactivation of MEK/ERK/MYC signaling. Nat Commun. 11:5463.

15. Jaiswal BS, Durinck S, Stawiski EW et al. (2018) ERK Mutations and Amplification Confer Resistance to ERK-Inhibitor Therapy. Clin Cancer Res. 24:4044-4055.

16. Jha S, Morris EJ, Hruza A et al. (2016) Dissecting Therapeutic Resistance to ERK Inhibition. Mol Cancer Ther. 15:548-559.

17. Duffy AG, Makarova-Rusher OV, Ulahannan SV et al. (2016) Modulation of tumor eIF4E by antisense inhibition: A phase I/II translational clinical trial of ISIS 183750-an antisense oligonucleotide against eIF4E-in combination with irinotecan in solid tumors and irinotecan-refractory colorectal cancer. Int J Cancer. 139:1648-1657.

18. Hong DS, Kurzrock R, Oh Y et al. (2011) A phase 1 dose escalation, pharmacokinetic, and pharmacodynamic evaluation of eIF-4E antisense oligonucleotide LY2275796 in patients with advanced cancer. Clin Cancer Res. 17:6582-6591.

19. Brandão M, Caparica R, Eiger D, de Azambuja E (2019) Biomarkers of response and resistance to PI3K inhibitors in estrogen receptor-positive breast cancer patients and combination therapies involving PI3K inhibitors. Ann Oncol. 30:x27-x42.

20. Wright SCE, Vasilevski N, Serra V, Rodon J, Eichhorn PJA (2021) Mechanisms of Resistance to PI3K Inhibitors in Cancer: Adaptive Responses, Drug Tolerance and Cellular Plasticity. Cancers (Basel). 13

21. Coleman N, Subbiah V, Pant S et al. (2021) Emergence of mTOR mutation as an acquired resistance mechanism to AKT inhibition, and subsequent response to mTORC1/2 inhibition. NPJ Precis Oncol. 5:99.

22. Lin A, Hu Q, Li C et al. (2017) The LINK-A lncRNA interacts with PtdIns(3,4,5)P_3_ to hyperactivate AKT and confer resistance to AKT inhibitors. Nat Cell Biol. 19:238-251.

23. Martorana F, Motta G, Pavone G et al. (2021) AKT Inhibitors: New Weapons in the Fight Against Breast Cancer. Front Pharmacol. 12:662232.

24. Sommer EM, Dry H, Cross D, Guichard S, Davies BR, Alessi DR (2013) Elevated SGK1 predicts resistance of breast cancer cells to Akt inhibitors. Biochem J. 452:499-508.

25. Antonuzzo L, Del Re M, Barucca V et al. (2017) Critical focus on mechanisms of resistance and toxicity of m-TOR inhibitors in pancreatic neuroendocrine tumors. Cancer Treat Rev. 57:28-35.

26. Faes S, Demartines N, Dormond O (2017) Resistance to mTORC1 Inhibitors in Cancer Therapy: From Kinase Mutations to Intratumoral Heterogeneity of Kinase Activity. Oxid Med Cell Longev. 2017:1726078.

27. Wagle N, Grabiner BC, Van Allen EM et al. (2014) Response and acquired resistance to everolimus in anaplastic thyroid cancer. N Engl J Med. 371:1426-1433.

28. Islam S, Espitia CM, Persky DO, Carew JS, Nawrocki ST (2020) Resistance to histone deacetylase inhibitors confers hypersensitivity to oncolytic reovirus therapy. Blood Adv. 4:5297-5310.

29. Lee JH, Choy ML, Marks PA (2012) Mechanisms of resistance to histone deacetylase inhibitors. Adv Cancer Res. 116:39-86.

30. Robey RW, Chakraborty AR, Basseville A et al. (2011) Histone deacetylase inhibitors: emerging mechanisms of resistance. Mol Pharm. 8:2021-2031.

31. Kuipers EJ, Grady WM, Lieberman D et al. (2015) Colorectal cancer. Nat Rev Dis Primers. 1:15065.

32. Carvalho B, Sillars-Hardebol AH, Postma C et al. (2012) Colorectal adenoma to carcinoma progression is accompanied by changes in gene expression associated with ageing, chromosomal instability, and fatty acid metabolism. Cell Oncol (Dordr). 35:53-63.

33. D’Abaco GM, Whitehead RH, Burgess AW (1996) Synergy between Apc min and an activated ras mutation is sufficient to induce colon carcinomas. Mol Cell Biol. 16:884-891.

34. Fearon ER, Vogelstein B (1990) A genetic model for colorectal tumorigenesis. Cell. 61:759-767.

35. Armaghany T, Wilson JD, Chu Q, Mills G (2012) Genetic alterations in colorectal cancer. Gastrointest Cancer Res. 5:19-27.

36. Jeong WJ, Ro EJ, Choi KY (2018) Interaction between Wnt/β-catenin and RAS-ERK pathways and an anti-cancer strategy via degradations of β-catenin and RAS by targeting the Wnt/β-catenin pathway. NPJ Precis Oncol. 2:5.

37. Lee SK, Hwang JH, Choi KY (2018) Interaction of the Wnt/β-catenin and RAS-ERK pathways involving co-stabilization of both β-catenin and RAS plays important roles in the colorectal tumorigenesis. Adv Biol Regul. 68:46-54.

38. Sansom OJ, Meniel V, Wilkins JA et al. (2006) Loss of Apc allows phenotypic manifestation of the transforming properties of an endogenous K-ras oncogene in vivo. Proc Natl Acad Sci U S A. 103:14122-14127.

39. Cha PH, Choi KY (2016) Simultaneous destabilization of β-catenin and Ras via targeting of the axin-RGS domain as a potential therapeutic strategy for colorectal cancer. BMB Rep. 49:455-456.

40. Clevers H (2013) The intestinal crypt, a prototype stem cell compartment. Cell. 154:274-284.

41. Anderson CB, Neufeld KL, White RL (2002) Subcellular distribution of Wnt pathway proteins in normal and neoplastic colon. Proc Natl Acad Sci U S A. 99:8683-8688.

42. Kobayashi M, Honma T, Matsuda Y et al. (2000) Nuclear translocation of beta-catenin in colorectal cancer. Br J Cancer. 82:1689-1693.

43. Araki K, Ogata T, Kobayashi M, Yatani R (1995) A morphological study on the histogenesis of human colorectal hyperplastic polyps. Gastroenterology. 109:1468-1474.

44. Wong WM, Mandir N, Goodlad RA et al. (2002) Histogenesis of human colorectal adenomas and hyperplastic polyps: the role of cell proliferation and crypt fission. Gut. 50:212-217.

45. Cristóbal I, Manso R, Rincón R et al. (2014) Phosphorylated protein phosphatase 2A determines poor outcome in patients with metastatic colorectal cancer. Br J Cancer. 111:756-762.

46. Cristóbal I, Manso R, Rincón R et al. (2014) PP2A inhibition is a common event in colorectal cancer and its restoration using FTY720 shows promising therapeutic potential. Mol Cancer Ther. 13:938-947.

47. Wu WK, Wang XJ, Cheng AS et al. (2013) Dysregulation and crosstalk of cellular signaling pathways in colon carcinogenesis. Crit Rev Oncol Hematol. 86:251-277.

48. Yan M, Li G, An J (2017) Discovery of small molecule inhibitors of the Wnt/β-catenin signaling pathway by targeting β-catenin/Tcf4 interactions. Exp Biol Med (Maywood). 242:1185-1197.

49. Germann UA, Furey BF, Markland W et al. (2017) Targeting the MAPK Signaling Pathway in Cancer: Promising Preclinical Activity with the Novel Selective ERK1/2 Inhibitor BVD-523 (Ulixertinib). Mol Cancer Ther. 16:2351-2363.

50. Sullivan RJ, Infante JR, Janku F et al. (2018) First-in-Class ERK1/2 Inhibitor Ulixertinib (BVD-523) in Patients with MAPK Mutant Advanced Solid Tumors: Results of a Phase I Dose-Escalation and Expansion Study. Cancer Discov. 8:184-195.

51. Ahronian LG, Sennott EM, Van Allen EM et al. (2015) Clinical Acquired Resistance to RAF Inhibitor Combinations in BRAF-Mutant Colorectal Cancer through MAPK Pathway Alterations. Cancer Discov. 5:358-367.

52. Carlino MS, Todd JR, Gowrishankar K et al. (2014) Differential activity of MEK and ERK inhibitors in BRAF inhibitor resistant melanoma. Mol Oncol. 8:544-554.

53. Hatzivassiliou G, Liu B, O’Brien C et al. (2012) ERK inhibition overcomes acquired resistance to MEK inhibitors. Mol Cancer Ther. 11:1143-1154.

54. Lalani AI, Moore CR, Luo C, Kreider… BZ (2015) Myeloid cell TRAF3 regulates immune responses and inhibits inflammation and tumor development in mice. The Journal of ….

55. Morris EJ, Jha S, Restaino CR et al. (2013) Discovery of a novel ERK inhibitor with activity in models of acquired resistance to BRAF and MEK inhibitors. Cancer Discov. 3:742-750.

56. Liu CY, Huang TT, Huang CT et al. (2017) EGFR-independent Elk1/CIP2A signalling mediates apoptotic effect of an erlotinib derivative TD52 in triple-negative breast cancer cells. Eur J Cancer. 72:112-123.

57. Yu HC, Hung MH, Chen YL et al. (2014) Erlotinib derivative inhibits hepatocellular carcinoma by targeting CIP2A to reactivate protein phosphatase 2A. Cell Death Dis. 5:e1359.

58. Shagufta, Ahmad I (2017) An insight into the therapeutic potential of quinazoline derivatives as anticancer agents. Medchemcomm. 8:871-885.

59. Martin LR, Williams SL, Haskard KB, DiMatteo MR (2005) The challenge of patient adherence. Therapeutics and Clinical Risk Management. 1:189.

60. Song M, Bode AM, Dong Z, Lee MH (2019) AKT as a Therapeutic Target for Cancer. Cancer Res. 79:1019-1031.

61. Phelps RA, Chidester S, Dehghanizadeh S et al. (2009) A two-step model for colon adenoma initiation and progression caused by APC loss. Cell. 137:623-634.

62. Bid HK, Roberts RD, Manchanda PK, Houghton PJ (2013) RAC1: an emerging therapeutic option for targeting cancer angiogenesis and metastasis. Mol Cancer Ther. 12:1925-1934.

63. Wu D, Pan W (2010) GSK3: a multifaceted kinase in Wnt signaling. Trends Biochem Sci. 35:161-168.

64. Shakoori A, Ougolkov A, Yu ZW et al. (2005) Deregulated GSK3beta activity in colorectal cancer: its association with tumor cell survival and proliferation. Biochem Biophys Res Commun. 334:1365-1373.

65. Wang HL, Hart J, Fan L, Mustafi R, Bissonnette M (2011) Upregulation of glycogen synthase kinase 3β in human colorectal adenocarcinomas correlates with accumulation of CTNNB1. Clin Colorectal Cancer. 10:30-36.

66. Wu Y, Deng J, Rychahou PG, Qiu S, Evers BM, Zhou BP (2009) Stabilization of snail by NF-kappaB is required for inflammation-induced cell migration and invasion. Cancer Cell. 15:416-428.

67. Cancer GAN (2012) Comprehensive molecular characterization of human colon and rectal cancer. Nature. 487:330-337.

68. Zhang L, Theodoropoulos PC, Eskiocak U et al. (2016) Selective targeting of mutant adenomatous polyposis coli (APC) in colorectal cancer. Sci Transl Med. 8:361ra140.
